# 14-3-3theta Protects against Neurotoxicity in a Cellular Parkinson's Disease Model through Inhibition of the Apoptotic Factor Bax

**DOI:** 10.1371/journal.pone.0021720

**Published:** 2011-07-20

**Authors:** Sunny R. Slone, Mathieu Lesort, Talene A. Yacoubian

**Affiliations:** 1 Department of Neurology, Center for Neurodegeneration and Experimental Therapeutics, University of Alabama at Birmingham, Birmingham, Alabama, United States of America; 2 Department of Psychiatry, University of Alabama at Birmingham, Birmingham, Alabama, United States of America; McGill University, Canada

## Abstract

Disruption of 14-3-3 function by alpha-synuclein has been implicated in Parkinson's disease. As 14-3-3s are important regulators of cell death pathways, disruption of 14-3-3s could result in the release of pro-apoptotic factors, such as Bax. We have previously shown that overexpression of 14-3-3θ reduces cell loss in response to rotenone and MPP^+^ in dopaminergic cell culture and reduces cell loss in transgenic *C. elegans* that overexpress alpha-synuclein. In this study, we investigate the mechanism for 14-3-3θ's neuroprotection against rotenone toxicity. While 14-3-3s can inhibit many pro-apoptotic factors, we demonstrate that inhibition of one factor in particular, Bax, is important to 14-3-3s' protection against rotenone toxicity in dopaminergic cells. We found that 14-3-3θ overexpression reduced Bax activation and downstream signaling events, including cytochrome C release and caspase 3 activation. Pharmacological inhibition or shRNA knockdown of Bax provided protection against rotenone, comparable to 14-3-3θ's neuroprotective effects. A 14-3-3θ mutant incapable of binding Bax failed to protect against rotenone. These data suggest that 14-3-3θ's neuroprotective effects against rotenone are at least partially mediated by Bax inhibition and point to a potential therapeutic role of 14-3-3s in Parkinson's disease.

## Introduction

Disruption of 14-3-3 expression and function has been implicated in the pathogenesis of Parkinson's disease (PD). This highly conserved protein family, which includes seven isoforms in mammals, are key regulators of cell death [1]. 14-3-3 proteins form homo- and heterodimers that create a concave groove in which ligands bind [2], [3]. Upon ligand binding, 14-3-3s can alter the conformational state of the ligand to alter activity or can bring together two ligands to interact [2], [3]. 14-3-3 ligands are implicated in many cellular functions, including transcription, metabolism, and apoptosis [2], [3]. In general, 14-3-3 isoforms act to promote cell survival through inhibition of many known pro-apoptotic factors [1].

In PD, several 14-3-3 isoforms – 14-3-3ε, γ, θ, and ζ – colocalize with the protein alpha-synuclein (α-syn) in Lewy bodies [4], [5]. Although its mechanism of toxicity is unclear, α-syn plays a central role in PD [6], [7], [8], [9], [10], [11], and the amount of 14-3-3s that coimmunoprecipitates with α-syn is increased in PD brains [12]. We have previously shown that the expression of several 14-3-3 isoforms is decreased in the brains of transgenic mice that overexpress wildtype human α-syn [13], [14]. Because of 14-3-3s' anti-apoptotic role, we have hypothesized that disruption of 14-3-3s by α-syn in PD could lead to the activation of pro-apoptotic pathways that are normally inhibited by 14-3-3s. In support of this hypothesis, we have shown that overexpression of 14-3-3θ, ε, or γ reduced cell loss in response to rotenone and MPP^+^ in dopaminergic cell culture, while other isoforms showed variable effects [14]. Human 14-3-3θ and the *C. elegans* 14-3-3 homologue *ftt-2* also reduced cell loss in transgenic *C. elegans* that overexpress α-syn [14]. The mechanism by which 14-3-3s are neuroprotective has not been examined in these PD models.

14-3-3s' effect on cell survival is thought to be mediated by their ability to inhibit pro-apoptotic factors. 14-3-3s have been demonstrated to bind and inhibit several different apoptotic factors, including BAD, Bax, and Bim [1], [15], [16], [17], [18]. Bax is an essential component in the apoptotic cascade, and its activation is induced by rotenone and MPTP, neurotoxins that are used to produce animal models of PD [19], [20], [21], [22]. In the pro-survival state, Bax is thought to be retained in the cytosol by binding to 14-3-3s. In response to pro-apoptotic signals, Bax can become dissociated from 14-3-3s and then be translocated to the mitochondria [16], [17], [23]. Similarly, other pro-apoptotic factors, such as BAD, can be bound by 14-3-3s to prevent the activation of apoptosis [24].

Here we investigate whether inhibition of Bax plays a role in 14-3-3s' neuroprotective effect against rotenone. Because the theta isoform showed the most substantial and consistent neuroprotection in our previous experiments, we focused on this isoform for the current study. We show that the rotenone-mediated Bax activation is inhibited when 14-3-3θ is overexpressed. Preventing Bax activation by alternative means imparts similar reduction in rotenone-induced cell death as 14-3-3θ overexpression, and disruption of 14-3-3θ's ability to bind Bax eliminates its protection against rotenone toxicity. These findings suggest that inhibition of Bax is key to 14-3-3θ's neuroprotective effects against rotenone.

## Methods

### 14-3-3θ cell lines

Full-length 14-3-3θ was subcloned into the expression vector pcDNA3.1/V5-His-TOPO (Invitrogen, Carlsbad, CA). C-terminally deleted 14-3-3θ (amino acids 1-239) was created by subcloning the DNA fragment representing amino acids 1 to 239 by PCR (forward primer 5′ caccgatcaaagtggtgggactcg; reverse primer 5′ cgcatcacattcttctcctg) into the expression vector pcDNA3.1D/V5-His-TOPO (Invitrogen). Both full-length and mutant 14-3-3θ were tagged with V5-His at the C-terminal end. SK-N-BE(2)-M17 (M17) cells (ATCC, Manassas, VA) were transfected with these constructs using Superfect (Qiagen, Germantown, MD), and stably-transfected cells were selected for in the presence of G418 (500 µg/ml; Invitrogen). Experimental controls included M17 cells transfected with the empty pcDNA3.1/V5-His vector and selected for stable transfection in the presence of G418.

### Immunoprecipitation

Control, 14-3-3θ, or 14-3-3θ mutant cell lysates were sonicated for 15 seconds on ice in lysis buffer [150 mM NaCl, 10 mM Tris-HCl, pH 7.4, 1 mM EDTA, 1 mM EGTA, protease and phosphatase inhibitor cocktail (Roche Diagnostics, Indianapolis, IN), 0.5% NP-40] and centrifuged at 14000 g for 10 minutes. Protein concentrations were determined by the bicinchoninic acid assay (Pierce, Rockford, IL). Lysates were precleared with Protein G Sepharose (Invitrogen) for one hour, and then incubated overnight at 4C with 2 µg of a rabbit polyclonal antibody against Bax (Cell Signaling, Danvers, MA) or rabbit IgG (Cell Signaling). After a 3 hour incubation of the antibody-lysate mix with Protein G Sepharose beads that had been preincubated with bovine serum albumin, Sepharose beads were washed in lysis buffer five times and then boiled for five minutes in 4xDTT sample loading buffer (0.25 M Tris-HCl, pH 6.8, 8% SDS, 200 mM DTT, 30% glycerol, bromophenol blue). Immunoprecipitate samples were analyzed by Western blotting.

### Subfractionation

Control and 14-3-3θ cells were resuspended into cavitation buffer (5 mM HEPES, pH 7.4, 3 mM MgCl_2_, 1 mM EGTA, 250 mM sucrose). Cells were then fractionated and mitochondria were isolated as previously described with minor modifications [25]. Briefly, cells were disrupted by nitrogen cavitation (250 psi) for five minutes on ice. After centrifugation of the resulting cell lysate at 5000 g for five minutes, the supernatant fraction was saved for preparation of the cytosolic fraction, while the pellet was saved for preparation of the mitochondrial fraction. The cytosolic fraction was obtained by centrifugation of the supernatant at 100000 g for one hour and concentration of the resulting supernatant using a SpeedVac concentrator. To obtain the mitochondrial fraction, the pellet from the initial centrifugation at 5000 g was resuspended in cavitation buffer and layered over a discontinuous 1.0/1.5M sucrose gradient prior to being centrifuged at 100000 g for 30 minutes at 4C. A hazy ring corresponding to the mitochondrial fraction was carefully recovered from the interface between the two sucrose solutions and diluted 1∶2 in 5 mM HEPES (pH 7.4), 3 mM MgCl_2_, and 1 mM EGTA prior to centrifugation at 20000 g for ten minutes at 4C. The resulting pellet was washed several times in cavitation buffer without sucrose, and then resuspended and sonicated in lysis buffer (175 mM NaCl, 50 mM Tris-HCl, pH 7.4, 5 mM EDTA, protease and phosphatase inhibitor cocktail, 1% Triton X-100). The resulting fractions were examined for Bax and cytochrome C by Western blotting.

### Bax oligomerization assay

Control and 14-3-3θ cells were collected in cavitation buffer and then disrupted by nitrogen cavitation (250 psi for 5 minutes) at 4C. The resulting cellular lysates were centrifugated at 300 g for three minutes at 4C to remove cellular debris. The resulting supernatant was centrifuged at 21000 g for 10 minutes at 4C. The mitochondrial-enriched pellet was washed three times in cavitation buffer and then solubilized in 2% CHAPS buffer for one hour at 4C. This mitochondrial-enriched sample was then crosslinked with 1 mM ethylene-glycol-bis(succinic acid N-hydroxy-succinimide ester) (Sigma) for 30 minutes at room temperature as previously described [26]. Protein samples were analyzed for Bax monomers and oligomers by Western blotting using a polyclonal antibody against Bax.

### Western Blot

Protein samples were resolved on 15% SDS-polyacrylamide gels and transferred to PVDF or nitrocellulose membranes. Blots were blocked in 5% non-fat dry milk in TBST (25 mM Tris-HCl, pH 7.6, 137 mM NaCl, 0.1% Tween-20) for one hour, and then incubated overnight with primary mouse monoclonal antibody against 14-3-3θ (1∶1000; Abcam, #ab10439, Cambridge, MA), rabbit polyclonal antibody against Bax (1∶1000; Cell Signaling, #2772), mouse monoclonal antibody against cytochrome C (1∶1000; Thermo Scientific, #MS-1192, Fremont, CA), rabbit polyclonal antibody against cleaved caspase 3 (1∶1000; Cell Signaling, #9661), mouse monoclonal antibody against cyclophilin D (1∶7500; EMD Biosciences, #AP1035, Gibbstown, NJ), mouse monoclonal antibody against the V5 epitope (1∶5000; Invitrogen, #R960), or mouse monoclonal antibody against α-tubulin (1∶1000; Sigma-Aldrich, #T9026, St. Louis, MO). After three washes in Tris-buffered saline with 0.1% Tween-20 (TBST), blots were incubated with HRP-conjugated goat anti-mouse or anti-rabbit secondary antibody (Jackson ImmunoResearch, West Grove, PA) for two hours and then washed in TBST six times for ten minutes each. Immunoreactive proteins were detecting using the enhanced chemiluminescence method (Pierce). Immunoblots were quantified using densitometry (Un-Scan-It Automated Digitizing System, Silk Scientific, Orem, UT).

### Immunocytochemistry

Cells were fixed in 2% paraformaldehyde for 15 minutes, washed in Tris-buffered saline (TBS), and incubated in 0.2% CHAPS buffer for 30 minutes. After an hour incubation in blocking buffer (2% goat serum, 3% bovine serum albumin in TBS), cells were incubated with the 6A7 mouse monoclonal antibody against Bax (1∶1500; Sigma-Aldrich, #B8429) overnight at 4C. After several washes in TBS, cells were incubated with a secondary Alexa 488-conjugated goat anti-mouse antibody (Invitrogen) for two hours at room temperature. After three washes in TBS, cells were incubated with 1 µg/ml Hoechst 33342 (Invitrogen) for five minutes, rinsed in TBS, and then mounted in Vectashield (Vector Laboratories, Burlingame, CA). Cells were imaged using a Nikon Eclipse E800 epifluorescence microscope, and images were captured with a Spot Flex CCD camera (Diagnostic Instruments, Sterling Heights, MI). Ten high power (20X) fields per well were randomly selected for quantification, and the number of 6A7-positive cells and the total number of cells stained by Hoechst 33342 were counted per high power field with the rater blind to experimental conditions. Four wells per condition were stained in each experiment, with a total of two independent experiments.

### JC-1 assay

Mitochondrial membrane potential (Δψm) was determined using the fluorescent dye 5,5,6,6-tetrachloro-1,1,3,3-tetraethylbenzimidazolyl-carbocyanine iodide (JC-1), which is able to enter mitochondria selectively [27]. JC-1 exists as a monomer emitting at 528 nm after excitation at 485 nm; however, depending on the Δψm, JC-1 is able to form aggregates that are associated with a large shift in emission (590 nm) after excitation at 530 nm. The color of JC-1 changes from green to red as the mitochondrial membrane becomes more polarized. The ratio of JC-1 aggregates to monomers is independent of cell number or mitochondrial density, such that quantification of the fluorescent state of JC-1 is a direct indicator of the relative mitochondrial potential. Cell staining was performed as follows: cells were plated in 24-well plates and treated with rotenone (Sigma-Aldrich) at varying concentrations. After drug treatment, cells were incubated with 5 µg/ml JC-1 for 30 minutes at 37C. After washing cells twice with PBS, fluorescence was measured using a fluorescent plate reader (Bio-Tek Synergy HT, Winooski, VT) set at excitation 485 nm and emission 528 nm for the green monomer, and at excitation 530 nm and emission 590 nm for the red aggregates. The ratio of JC-1 fluorescence (590 nm/ 528 nm) was calculated as a direct measure of Δψm.

### LDH assay

Cells were grown in pyruvate-free DMEM for a few days prior to plating in 24-well collagen-treated plates. Following treatment with rotenone in serum-free DMEM for 48 hours, toxicity was assayed by lactate dehydrogenase (LDH) release into media using the LDH cytotoxicity kit (Roche). LDH release into media was normalized to maximal LDH release for each well.

### Statistical analysis

GraphPad Prism 5 (La Jolla, CA) was used for statistical analysis of experiments. Western blots, 6A7 immunostaining, JC-1 assay, and LDH assay experiments were analyzed by 2-way ANOVA, followed by post-hoc Bonferroni's multiple comparison test. For the Bax subfractionation experiment, Bax expression level for rotenone-treated cells was normalized to Bax expression level for corresponding untreated cells. Normalized Bax levels were analyzed by one sample t-test.

## Results

### 14-3-3θ immunoprecipitates with Bax

**Figure 1:**
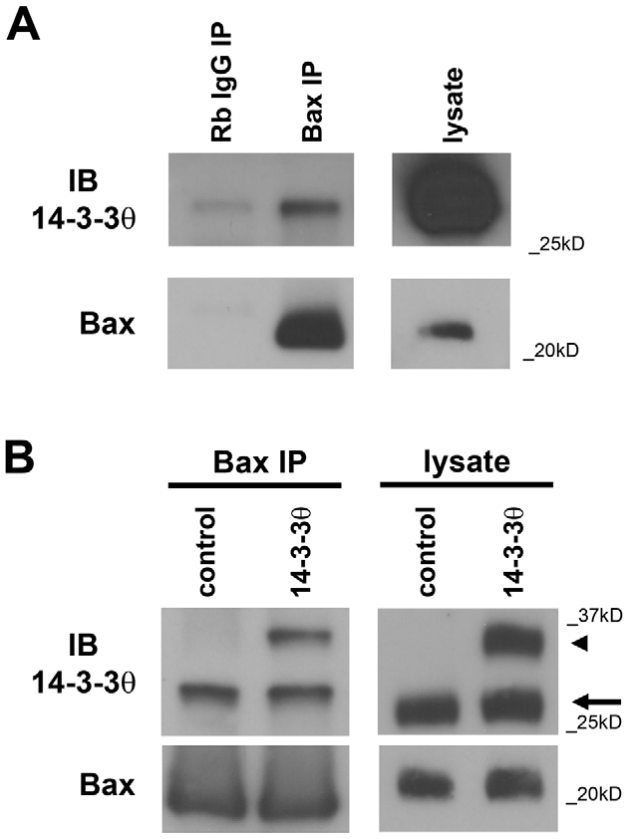
14-3-3θ immunoprecipitates with Bax in M17 dopaminergic cells. **a)** Cell lysates from M17 cells were immunoprecipitated with a polyclonal rabbit antibody against Bax or rabbit IgG, and resulting immunoprecipitants were blotted with a monoclonal mouse antibody against 14-3-3θ in top blot. Lysate lane on right is shown at a different exposure time than the immunoprecipitant lanes from the same gel. Blot was reprobed with anti-Bax antibody to verify Bax pulldown (bottom blot). 14-3-3θ shows specific immunoprecipitation with Bax. **b)** Cell lysates from M17 cells stably transfected with empty vector or 14-3-3θ tagged with the V5 epitope tag were immunoprecipitated with a polyclonal antibody against Bax and then immunoblotted against 14-3-3θ. Both endogenous 14-3-3θ (lower band marked by arrow) and exogenous, tagged 14-3-3θ (higher band marked by arrowhead) were immunoprecipitated with Bax from cells overexpressing 14-3-3θ, and the total amount of 14-3-3θ immunoprecipitated was increased in 14-3-3θ cells compared to empty vector control cells. Lysate lanes on right were run on a separate gel from the immunoprecipitant lanes. Blot was reprobed with anti-Bax antibody to verify pulldown of Bax (bottom blot).

We have previously shown that overexpression of 14-3-3θ in the dopamine-producing cell line SK-N-BE(2)-M17 (M17) reduces cell death induced by the neurotoxin rotenone [14]. To investigate whether Bax inhibition may play a role in 14-3-3θ's neuroprotective effects, we first examined whether 14-3-3θ can immunoprecipitate with Bax in M17 cells. After immunoprecipitation of lysates from control M17 cells with a polyclonal antibody against Bax or rabbit IgG, immunoprecipitates were immunoblotted with a monoclonal antibody against 14-3-3θ. Immunoprecipitation revealed that 14-3-3θ did interact specifically with Bax in control cells (Fig. 1a). We next examined whether 14-3-3θ immunoprecipitated with Bax in M17 cells that stably overexpressed 14-3-3θ tagged with V5-His. Lysates from vector control and 14-3-3θ cells were immunoprecipitated with an anti-Bax antibody and then immunoblotted with an anti-14-3-3θ antibody. Endogenous 14-3-3θ (Fig. 1b; lower band around 30 kD, arrow) was immunoprecipitated with Bax in both vector control and 14-3-3θ stable cells, while exogenous, tagged 14-3-3θ was immunoprecipitated with Bax only in 14-3-3θ stable cells (Fig. 1b; higher band around 35 kD, arrowhead). Thus, 14-3-3θ immunoprecipitates with Bax, and overexpression of 14-3-3θ results in an increase of its association with Bax.

### 14-3-3θ overexpression reduces Bax activation

**Figure 2:**
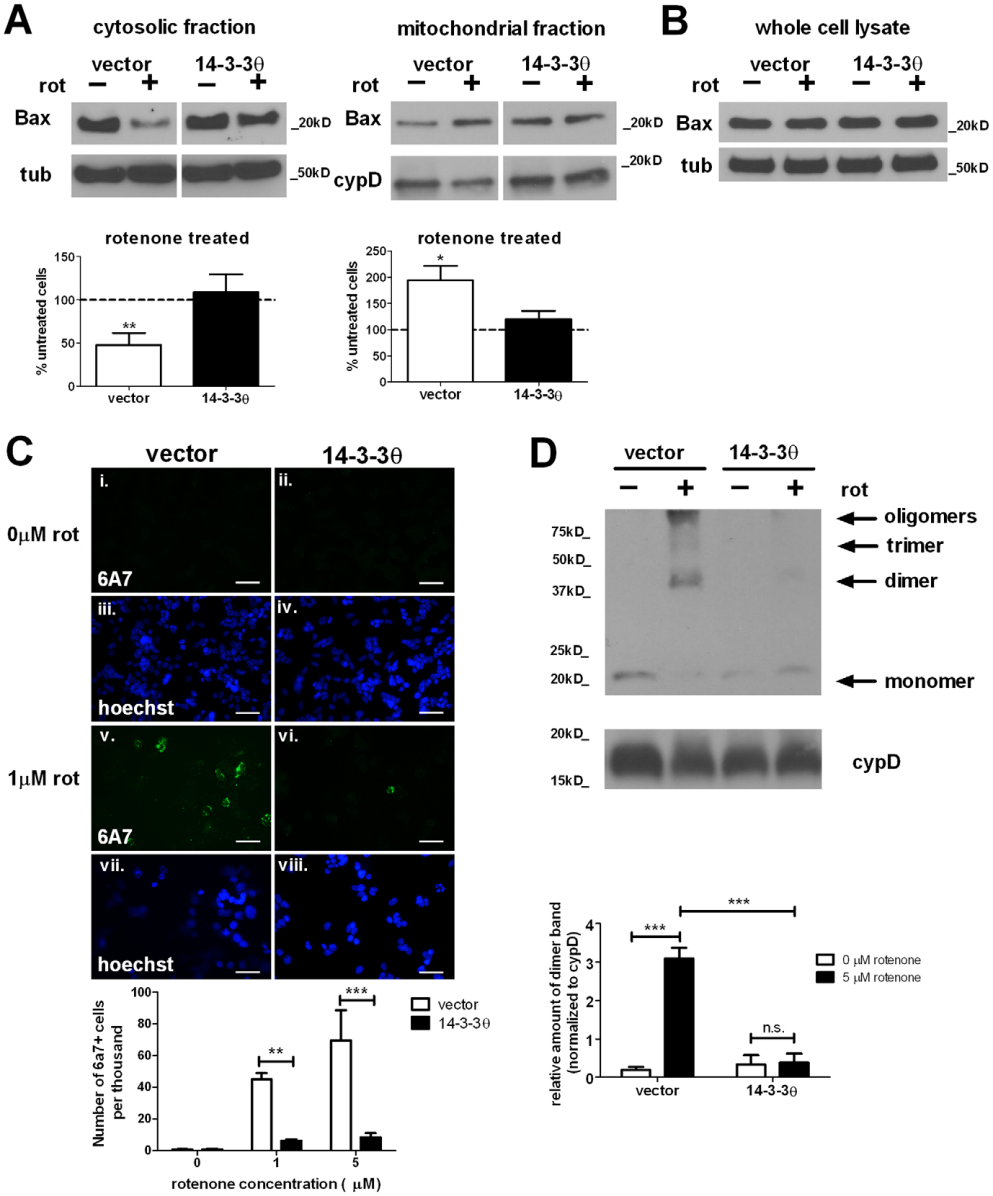
Rotenone-induced Bax activation is reduced in 14-3-3θ-overexpressing cells. **a) Less Bax translocated to mitochondria in 14-3-3θ cells in response to rotenone.** After treatment with 5 µM rotenone for 24 hours, vector control and 14-3-3θ cell lysates were subfractionated into cytosolic and mitochondrial fractions and immunoblotted with a polyclonal rabbit antibody against Bax. For each fraction, lanes for vector control and 14-3-3θ cells are from the same gel and exposure time but are separated for clarity with regard to quantification. Bax levels were normalized to tubulin for the cytosolic fraction or cyclophilin D for the mitochondrial fraction. Bax levels for rotenone-treated cells are shown as the relative percentage of the corresponding untreated cells. Densitometric quantification included seven separate experiments. Error bars reflect SEM. *p<0.05, **p<0.01 (one sample t-test). **b) Total Bax levels were unchanged with rotenone treatment in either cell line.** After treatment with 5 µM rotenone for 24 hours, whole cell lysates were immunoblotted with an anti-Bax antibody. **c) Fewer 14-3-3θ cells were positive for activated Bax upon rotenone treatment.** After treatment without (i-iv) or with rotenone (v-viii) for 16 hours, vector control and 14-3-3θ cells were fixed in 2% paraformaldehyde and immunostained with a monoclonal mouse antibody against the active Bax conformation (6A7) and a goat Alexa 488-conjugated anti-mouse secondary antibody (i, ii, v, vi). Nuclei were stained with Hoechst 33342 (iii, iv, vii, viii). The number of 6A7-positive cells was quantitated with rater blind to experimental conditions. Error bars reflect SEM. **p<0.01, ***p<0.001 (Bonferroni's multiple comparison test). Scale bar  = 50 µm. **d) Rotenone-induced Bax oligomerization was reduced in 14-3-3θ cells.** Vector control and 14-3-3θ stable cells were treated with 5 µM rotenone for 24 hours. Mitochondrially-enriched fractions were crosslinked and immunoblotted for oligomers with an anti-Bax antibody. Cyclophilin D served as loading control. Densitometric quantification includes three independent experiments. Error bars reflect SEM. ***p<0.001 (Bonferroni's multiple comparison test). n.s.  =  non-significant.

Since 14-3-3θ interacts with Bax as demonstrated by immunoprecipitation, we hypothesized that increased binding of Bax in the presence of higher 14-3-3θ levels could reduce Bax activation. In response to an apoptotic trigger, Bax undergoes conformational alterations and translocates to the mitochondrial outer membrane, where it undergoes oligomerization that leads to pore formation [28], [29]. We first tested whether 14-3-3θ overexpression altered the translocation of Bax into the mitochondria in response to rotenone. Control and 14-3-3θ stable cells were treated with 5 µM rotenone for approximately 24 hours, and then cell lysates were fractionated into cytosolic and mitochondrial fractions. For vector control cells treated with rotenone, immunoblotting of these fractions showed that Bax levels decreased to 48% of untreated cells in the cytosolic fraction (p<0.01, one sample t-test) and increased to 194% of untreated cells in the mitochondrial fraction (p<0.05, one sample t-test); this suggests that Bax translocates to the mitochondria upon rotenone treatment (Fig. 2a). In contrast, incubation of 14-3-3θ cells with rotenone did not result in significant changes in the cytosolic or mitochondrial Bax levels as determined by Western blotting (Fig. 2a). Levels of total Bax protein were similar in control and 14-3-3θ cell lines and were not affected by rotenone treatment in both cell lines, as determined by Western blotting of whole cell lysates (Fig. 2b).

We next examined whether rotenone induced Bax conformational changes and whether these changes were altered in the presence of 14-3-3θ overexpression. We did immunocytochemistry against activated Bax using the monoclonal antibody 6A7 that detects an N-terminal Bax epitope that is exposed only upon Bax activation [30], [31]. Vector control and 14-3-3θ stable cells were incubated in the absence or presence of 1 or 5 µM rotenone for 24 hours, and then cells were fixed and stained with the 6A7 antibody. As seen in Fig. 2c, the numbers of 6A7-positive cells increased with rotenone treatment in vector control cells, but this increase was significantly attenuated in 14-3-3θ cells (p<0.01 at 1 µM, p<0.001 at 5 µM, Bonferroni's multiple comparison test).

We then tested whether the formation of Bax oligomers is affected by 14-3-3θ overexpression. Vector control and 14-3-3θ cells were treated with 5 µM rotenone for 24 hours, and then mitochondrial-enriched cell lysates were crosslinked with 1 mM ethylene-glycol-bis(succinic acid N-hydroxy-succinimide ester), as previously described [26]. After crosslinking, lysates were run on a gel and immunoblotted with an antibody against Bax to detect monomers and oligomers of Bax. As seen in Fig. 2d, rotenone induced oligomerization of Bax in vector control cells, while 14-3-3θ cells showed a reduced level of Bax oligomers in response to rotenone treatment. The rotenone-mediated Bax dimer formation in 14-3-3θ cells was 15% of that in vector control cells treated with rotenone (p<0.001, Bonferroni's multiple comparison test). Therefore, Bax activation is reduced upon rotenone treatment in 14-3-3θ cells, as determined by decreased translocation, conformational change, and oligomerization.

### 14-3-3θ overexpression reduces signaling events downstream of Bax activation

**Figure 3:**
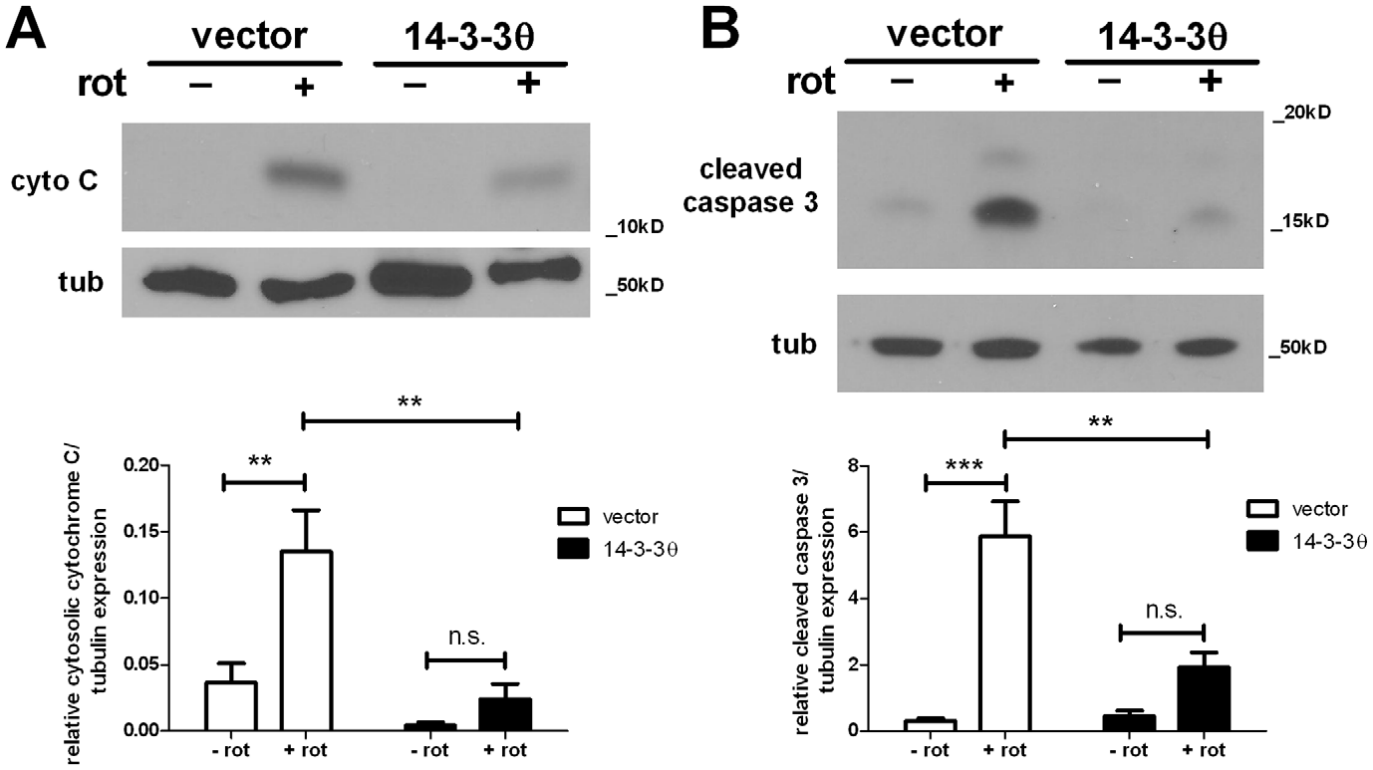
14-3-3θ overexpression reduces rotenone-induced cytochrome C release and caspase 3 cleavage. **a)** Vector control and 14-3-3θ cells were treated with 5 µM rotenone for 24 hours, and cytosolic fractions were immunoblotted with a mouse monoclonal antibody against cytochrome C. Densitometric quantification includes five independent experiments. Tubulin was used as the loading control for Western blots. Error bars reflect SEM. **p<0.01 (Bonferroni's multiple comparison test). n.s.  =  non-significant. **b)** Vector control and 14-3-3θ cells were treated with 5 µM rotenone for 24 hours, and cell lysates were immunoblotted with a rabbit polyclonal antibody against cleaved caspase 3. Densitometric quantification includes three independent experiments. Tubulin was used as the loading control for Western blots. Error bars reflect SEM. **p<0.01, ***p<0.001 (Bonferroni's multiple comparison test). n.s.  =  non-significant.

We next investigated whether apoptotic signaling events downstream of Bax were altered in 14-3-3θ cells upon rotenone treatment. Upon activation and oligomerization, Bax causes permeabilization of the outer mitochondrial membrane that results in cytochrome C release and caspase 3 activation. We examined whether cytochrome C release into the cytoplasm and caspase 3 activation were affected by 14-3-3θ overexpression by Western blotting. Cytochrome C release into the cytoplasm was observed in vector control cells upon rotenone (5 µM) treatment for 24 hours but was robustly decreased in 14-3-3θ cells. Cytochrome C release in 14-3-3θ cells was about 18% of that of vector control cells (Fig. 3a; p<0.01, Bonferroni's multiple comparison test). As shown by Western blotting of whole cell lysates, rotenone-mediated caspase 3 activation was also reduced in 14-3-3θ cells compared to vector control cells; quantitative analysis revealed that cleaved caspase 3 levels in 14-3-3θ cells was reduced to 33% of vector control cells (Fig. 3b; p<0.01, Bonferroni's multiple comparison test). Therefore, 14-3-3θ overexpression not only reduced Bax activation but also reduced the activation of signaling events downstream of its activation.

### Bax inhibition by alternate means is neuroprotective

**Figure 4:**
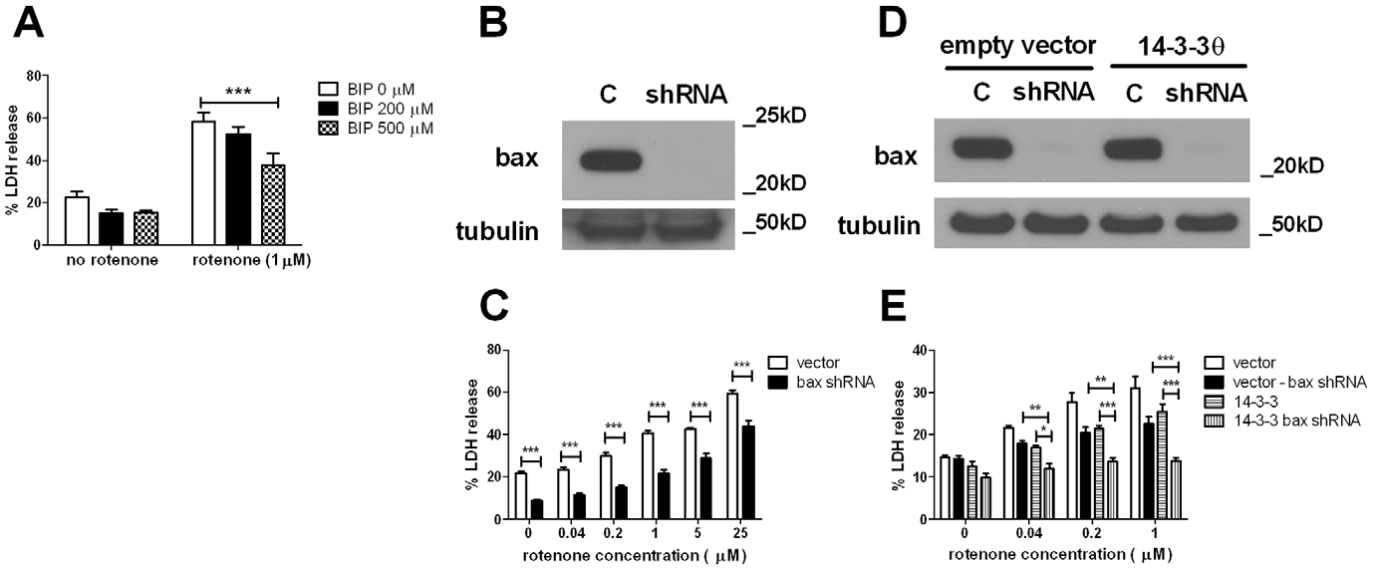
Bax inhibition through alternative means is protective against rotenone toxicity. **a)** M17 cells were pretreated with BIP (0, 200, or 500 µM) for four hours prior to treatment with rotenone at 1 µM. After 48 hours, cell death was assessed by LDH release into the culture media. LDH release into media was normalized to maximal LDH release for each well. Cells treated with BIP were more resistant to rotenone compared to untreated cells. Error bars reflect SEM. Results reflect three independent experiments with at least two replicates per experiment. ***p<0.001 (Bonferroni's multiple comparison test). **b)** shRNA targeting Bax showed considerable knockdown of Bax protein expression. Naïve M17 cells were infected with a pLKO.1 lentiviral construct containing Bax-specific shRNA sequence or with an empty pLKO.1 lentiviral construct (with no shRNA sequence; C). Infected cells were selected for in the presence of puromycin. Protein lysates from these infected cells were immunoblotted with a polyclonal antibody against Bax (top blot). Immunoblotting against tubulin (bottom blot) shows comparable protein loading. **c)** pLKO.1 control or Bax-shRNA M17 cells were treated with rotenone at varying concentrations for 48 hours. Cell death was assessed by LDH release. Bax-knockdown cells showed considerable protection against rotenone compared to control cells at all concentrations tested. Error bars reflect SEM. Results reflect three independent experiments with at least two replicates per experiment. ***p<0.001 (Bonferroni's multiple comparison test). **d)** shRNA targeting Bax also showed knockdown of Bax protein in both empty vector control and 14-3-3θ stable cell lines. Control and 14-3-3θ stable lines were infected with an empty pLKO.1 virus (C) or with the Bax-specific shRNA lentivirus. Protein lysates from these cells were immunoblotted with a polyclonal antibody against Bax and tubulin. **e)** Empty vector stable and 14-3-3θ stable cells infected with either empty pLKO.1 or Bax shRNA viruses were treated with rotenone at varying concentrations for 48 hours, and cell death was assessed by LDH release. Knockdown of Bax in 14-3-3θ stable cells provided additional reduction of rotenone toxicity. Error bars reflect SEM. Results reflect four independent experiments with at least two replicates per experiment. *p<0.05, **p<0.01, ***p<0.001 (Bonferroni's multiple comparison test).

If the inhibition of Bax by 14-3-3θ is sufficient to mediate 14-3-3θ's neuroprotective effects, then we predict that inhibition of Bax through alternative mechanisms should also reduce toxicity in response to rotenone in M17 cells. To test this, we inhibited Bax through two methods: 1) pharmacological inhibition with the Bax inhibitor peptide (BIP) [32], and 2) shRNA-mediated knockdown of Bax expression. Vector control and 14-3-3θ cells were pretreated with 200 or 500 µM BIP for four hours and then treated with 1 µM rotenone for 48 hours in the presence of BIP. Cell death was assayed by lactate dehydrogenase (LDH) release into the media, and LDH release was normalized to total LDH release per well, as previously described. BIP showed a reduction in rotenone toxicity in M17 cells in a dose-dependent manner. Treatment with 500 µM BIP reduced LDH release to 65% of cells treated with rotenone only (Fig. 4a; p<0.001, Bonferroni's multiple comparison test).

**Figure 5:**
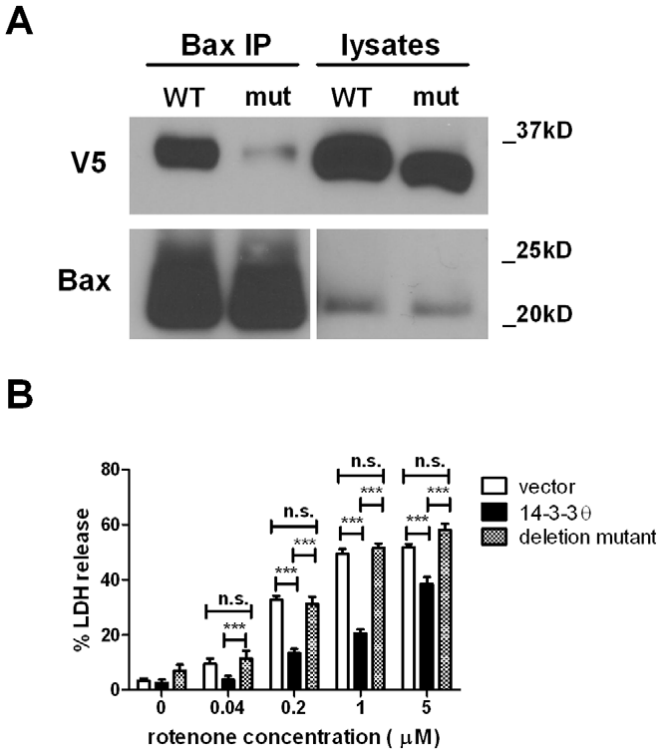
14-3-3θ mutant that cannot bind Bax is not protective against rotenone. **a)** Lysates from stable cells overexpressing either full-length 14-3-3θ or a C-terminally deleted mutant 14-3-3θ (aa1-239) were immunoprecipitated with a polyclonal rabbit antibody against Bax and then immunoblotted with a monoclonal mouse antibody against V5. Blot was reprobed with anti-Bax antibody to verify Bax pulldown (bottom blot). Lysate lanes on right in the Bax blot are shown at a different exposure time than the immunoprecipitant lanes from the same gel. Considerably much less mutant 14-3-3θ was immunoprecipitated with Bax compared to full-length 14-3-3θ. **b)** Vector control, full-length 14-3-3θ, or mutant 14-3-3θ cells were treated with rotenone for 48 hours. Cell death was assessed by LDH release. While full-length 14-3-3θ cells showed decreased cell death in response to rotenone, cells overexpressing mutant 14-3-3θ showed no protection against rotenone compared to vector control cells. Error bars reflect SEM. Results reflect three independent experiments with at least two replicates per experiment. ***p<0.001 (Bonferroni's multiple comparison test). n.s.  =  non-significant.

We then examined whether knockdown of Bax by shRNA would affect the sensitivity of M17 cells to rotenone. M17 cells were infected with lentivirus containing a shRNA targeting Bax or with lentivirus containing the pLKO.1 empty vector only. Cells infected with the Bax shRNA showed knockdown of Bax expression by Western blotting (Fig. 4b). After incubation with 1 µM rotenone for 48 hours, Bax shRNA cells showed reduced cell death compared to control cells, as measured by LDH release. At 1 µM rotenone, Bax knockdown reduced cell death to 54% of control cells (Fig. 4c; p<0.001, Bonferroni's multiple comparison test). The amount of protection with Bax knockdown was comparable to protection provided by 14-3-3θ overexpression that we have previously observed ([14] and Fig. 5b).

We next tested whether knockdown of Bax in 14-3-3θ stable cells would provide additional protection against rotenone. Both control stable and 14-3-3θ stable cell lines infected with Bax shRNA showed knockdown of Bax as determined by Western blotting (Fig. 4d). After incubation with rotenone for 48 hours, 14-3-3θ cells in which Bax expression was eliminated by shRNA showed additional protection against rotenone compared to 14-3-3θ cells infected with control (pLKO.1 empty vector) virus (Fig. 4e). Control stable cells infected with Bax shRNA showed less protection compared to 14-3-3θ cells infected with Bax shRNA (Fig. 4e).

### Mutant 14-3-3θ that cannot bind Bax is not protective against rotenone

To test whether the interaction of 14-3-3θ with Bax is required for its neuroprotective effects, we created a mutant 14-3-3θ that shows significantly reduced affinity for Bax. Upon apoptotic stimuli, caspases can cleave 14-3-3θ at Asp 239 in the C-terminal end that results in a reduction of 14-3-3θ's binding to Bax [16]. Unlike other 14-3-3 ligands, Bax binds to 14-3-3s in a phosphorylation-independent manner requiring the C-terminal end of 14-3-3 [16]. Our mutant has a deletion of six amino acids at the C-terminal end to mimic this caspase cleavage product. After creating a V5 epitope-tagged deletion mutant, we transfected M17 cells with this mutant construct and selected for stably-transfected cells.

We confirmed that this 14-3-3θ mutant does not immunoprecipitate with Bax in these stable 14-3-3θ mutant cells in contrast to full-length 14-3-3θ(Fig. 5a). We next investigated whether stable cells overexpressing the mutant 14-3-3θ were similarly resistant to rotenone treatment as were full-length 14-3-3θ cells. Control, full-length 14-3-3θ, and mutant 14-3-3θ cells were treated with varying doses of rotenone for 48 hours, and cell death was assayed by LDH release into the media. While 14-3-3θ cells showed reduction in cell death in response to rotenone treatment, cells overexpressing the mutant 14-3-3θ did not show any protection against rotenone toxicity (Fig. 5b).

### 14-3-3θ overexpression also protects against disruption of the mitochondrial membrane potential

**Figure 6:**
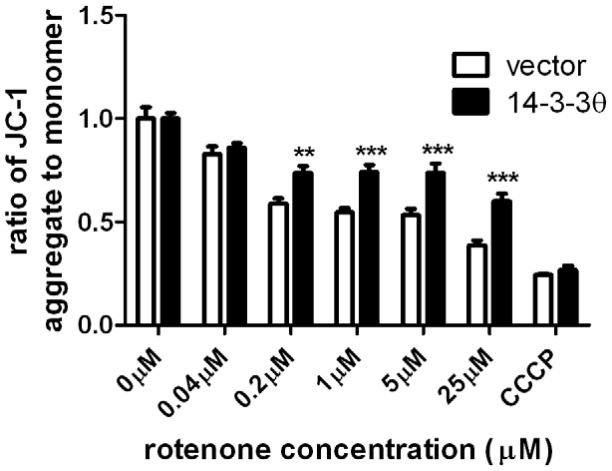
14-3-3θ overexpression reduces rotenone-induced disruption of mitochondrial membrane potential. Vector control and 14-3-3θ cells were treated with varying doses of rotenone or 10 µM carbonyl cyanide 3-chlorophenylhydrazone (CCCP), a mitochondrial toxin, for 24 hours. Mitochondrial membrane potential was assayed by the JC-1 assay. Ratio of aggregated JC-1 (red) to monomer JC-1 (green) for each condition was normalized to that ratio for the corresponding untreated cells. Results reflect three independent experiments with three replicates per experiment. Error bars reflect SEM. **p<0.01, ***p<0.001 (Bonferroni's multiple comparison test).

Our Bax knockdown experiments implicate additional mechanisms besides Bax inhibition for 14-3-3θ's neuroprotective effects. Since rotenone can directly inhibit Complex I to disrupt the mitochondrial membrane potential (Δψm), 14-3-3θ could modulate this effect in a manner independent of Bax inhibition. We measured the effect of 14-3-3θ overexpression on Δψm. Changes in Δψm were determined by using the ratiometric fluorescent dye JC-1. As expected, Δψm was reduced with increasing doses of rotenone in vector control cells. The rotenone-mediated decrease in Δψm was significantly attenuated in 14-3-3θ cells (Fig. 6). The mitochondrial respiratory chain uncoupler, carbonyl cyanide 3-chlorophenylhydrazone (CCCP) was used as a positive control for disruption of Δψm (Fig. 6).

## Discussion

In this study, we have investigated the mechanism by which 14-3-3θ is neuroprotective in a cellular PD model. While 14-3-3s are known to interact with and inhibit many pro-apoptotic factors, here we demonstrate that inhibition of one such factor in particular, Bax, is important to 14-3-3θ's protection against rotenone-induced toxicity in dopaminergic cells. We found that overexpression of 14-3-3θ reduced Bax activation and downstream signaling events, including cytochrome C release and caspase 3 activation. Inhibition of Bax by pharmacological or shRNA knockdown provided protection against rotenone toxicity, while 14-3-3θ mutant that is incapable of binding Bax showed no protection against rotenone toxicity. These findings suggest that Bax inhibition is required for 14-3-3θ's effects against rotenone. However, as 14-3-3θ overexpression promotes additional neuroprotection in the presence of Bax knockdown, additional mechanisms likely contribute to 14-3-3θ's neuroprotective effects. In this study, we focused on 14-3-3θ as it showed the most significant protection against cell death in several PD models [14]. As both 14-3-3ε and ζ can also interact with Bax [16], [23], it is possible that the neuroprotective effects of these isoforms seen in our previous study [14] could also be mediated through Bax inhibition.

Nomura *et al*. (2003) have previously shown that 14-3-3θ inhibits Bax by binding directly to Bax at the N- and C-terminal ends of Bax [16]. This interaction between 14-3-3s and Bax does not involve the usual phosphorylation binding motifs (RSXpSXP or RXXXpSXP) that most ligands require for binding to 14-3-3s. Cleavage of 14-3-3θ's C-terminal end by caspases results in the release of Bax [16], and we demonstrate that this 14-3-3θ cleavage product cannot protect against rotenone toxicity. The simplest interpretation of our data is that 14-3-3θ directly binds to Bax via its C-terminal end to induce neuroprotection. However, we cannot rule out the possibility that 14-3-3θ interacts indirectly with Bax by means of an intermediary ligand.

While our study reveals that inhibition of Bax is key to 14-3-3θ's neuroprotective effects, it is likely that other mechanisms do contribute to 14-3-3θ's effects. Knockdown of Bax in M17 cells did provide comparable protection against rotenone as 14-3-3θ overexpression did, but we found that knockdown of Bax in the presence of 14-3-3θ overexpression provided additional protection. This finding suggests that the amount of 14-3-3θ overexpression was likely not sufficient to inhibit all Bax expressed in our cells. It also suggests that additional mechanisms may also mediate 14-3-3θ's effects, as Bax knockdown in 14-3-3θ stable cells was more protective against rotenone than Bax knockdown in control stable cells. We did find that 14-3-3θ overexpression reduces disruption of Δψm by rotenone, suggesting that 14-3-3θ can act directly or indirectly at the mitochondrial respiratory chain in a manner that may be independent of Bax.

Given 14-3-3s' known interactions with other apoptotic factors [1], 14-3-3θ could also inhibit other pro-apoptotic factors to promote cell survival. We did investigate a potential role for BAD, another apoptotic factor inhibited by 14-3-3s, but we saw a comparable increase in BAD phosphorylation and translocation of BAD to the cytosol upon rotenone treatment in both control and 14-3-3θ cells (data not shown). Pro-apoptotic signals typically promote BAD dephosphorylation, resulting in release from 14-3-3 binding, mitochondrial translocation, and activation of apoptotic signaling cascades [33]. Since we observed decreased BAD activation, with increased BAD phosphorylation and decreased mitochondrial translocation in response to rotenone, we concluded that BAD is unlikely to play a role in our cellular system. Whether inhibition of other pro-apoptotic factors by 14-3-3θ contributes to its neuroprotective effects, inhibition of Bax is central to 14-3-3θ's neuroprotection, as disruption of the interaction between 14-3-3θ and Bax abolishes 14-3-3θ's effect.

Accumulating evidence supports the role of 14-3-3s in the pathogenesis of PD, and direct interactions with several other proteins implicated in PD has been shown. 14-3-3s have been demonstrated to colocalize with α-syn in Lewy Bodies and show increased immunoprecipitation with α-syn in PD brains [4], [12]. In addition, we have previously shown that expression levels of several 14-3-3 isoforms are reduced in transgenic mice overexpressing human wildtype α-syn [13], [14]. 14-3-3η can regulate the ubiquitin ligase activity of parkin, while disease-causing mutations in parkin disrupt its interaction with 14-3-3θ [12]. The interaction of 14-3-3s with LRRK2 suggests a role for 14-3-3s in regulating LRRK2 function, especially since some common disease-causing mutations in LRRK2 can disrupt its interaction with 14-3-3s [34], [35], [36].

While we do not directly examine the interaction of 14-3-3θ and Bax in a genetic model of PD, our data presented here has implications for the pathophysiology of PD. Rotenone is a widely accepted toxin-based model of PD. Pesticides have been associated with an increased risk of developing PD [37], [38], [39], [40], and oxidative stress has long been implicated in the pathogenesis of PD [41]. Rotenone is a pesticide that causes toxicity in cultured neuroblastoma cells via inhibition of complex I in mitochondria, and causes specific loss of nigral neurons in rodents when injected systemically [42], [43], [44]. Chronic treatment with rotenone causes α-syn aggregation *in vitro* and *in vivo* [42], [43], [44]. If increased α-syn levels observed in PD can cause disruption of 14-3-3 function, we predict that pro-apoptotic factors normally inhibited by 14-3-3s could be freed to activate cell death pathways that then contribute to the neurodegeneration seen in PD. Our data here supports this hypothesis. In response to rotenone, which induces α-syn aggregation in M17 cells [14], Bax is activated and cell death occurs. With overexpression of 14-3-3θ, Bax activation is reduced, resulting in decreased cell death in response to rotenone. In contrast, overexpression of mutant 14-3-3θ which cannot bind Bax is incapable of preventing rotenone toxicity. The balance of 14-3-3s and pro-apoptotic factors likely determines the fate of cells in response to rotenone toxicity. Further examination of this interaction between Bax and 14-3-3θ in other PD models will be of interest.

Bax has been previously investigated for its potential role in PD. Bax immunoreactivity is increased in the substantia nigra of PD brains compared to control [45], and its expression and activation are induced in cells treated with rotenone and MPP^+^ [19], [20], [22]. In a chronic MPTP mouse model, protein levels of Bax are increased within days after the last MPTP injection, and mitochondrial translocation of Bax is observed [21], [46]. Bax knockout mice are resistant to MPTP-induced neurodegeneration in the substantia nigra [21], while blocking the increase in Bax levels or blocking its mitochondrial translocation via other means can also reduce dopaminergic cell loss in MPTP-treated mice [47].

Our studies here show that 14-3-3θ is neuroprotective in a PD model through its inhibition of Bax. We show that M17 cells overexpressing 14-3-3θ show reduced Bax activation, signaling downstream of Bax, and cell death, while a mutant 14-3-3θ that cannot interact with Bax fails to reduce rotenone toxicity. As Bax activation is important to PD pathogenesis, 14-3-3θ could serve as a potential target for the development of new PD therapeutics.
